# Homozygosity Mapping in Leber Congenital Amaurosis and Autosomal Recessive Retinitis Pigmentosa in South Indian Families

**DOI:** 10.1371/journal.pone.0131679

**Published:** 2015-07-06

**Authors:** Sundaramurthy Srilekha, Tharigopala Arokiasamy, Natarajan N. Srikrupa, Vetrivel Umashankar, Swaminathan Meenakshi, Parveen Sen, Suman Kapur, Nagasamy Soumittra

**Affiliations:** 1 SNONGC Department of Genetics and Molecular Biology, Vision Research Foundation, Chennai, India; 2 Ph.D Scholar, Birla Institute of Technology & Science (BITS), Hyderabad, India; 3 Head, Centre for Bioinformatics, Vision Research Foundation, Chennai, India; 4 Department of Peadiatric ophthalmology and starbismus, Medical Research Foundation, Chennai, India; 5 Department of Vitreo-Retinal Services, Medical Research Foundation, Chennai, India; 6 Head, Department of Biological Science, Birla Institute of Technology & Science (BITS), Hyderabad, India; Hadassah-Hebrew University Medical Center, ISRAEL

## Abstract

Leber congenital amaurosis (LCA) and retinitis pigmentosa (RP) are retinal degenerative diseases which cause severe retinal dystrophy affecting the photoreceptors. LCA is predominantly inherited as an autosomal recessive trait and contributes to 5% of all retinal dystrophies; whereas RP is inherited by all the Mendelian pattern of inheritance and both are leading causes of visual impairment in children and young adults. Homozygosity mapping is an efficient strategy for mapping both known and novel disease loci in recessive conditions, especially in a consanguineous mating, exploiting the fact that the regions adjacent to the disease locus will also be homozygous by descent in such inbred children. Here we have studied eleven consanguineous LCA and one autosomal recessive RP (arRP) south Indian families to know the prevalence of mutations in known genes and also to know the involvement of novel loci, if any. Complete ophthalmic examination was done for all the affected individuals including electroretinogram, fundus photograph, fundus autofluorescence, and optical coherence tomography. Homozygosity mapping using Affymetrix 250K HMA GeneChip on eleven LCA families followed by screening of candidate gene(s) in the homozygous block identified mutations in ten families; *AIPL1* – 3 families, *RPE65*- 2 families, *GUCY2D*, *CRB1*, *RDH12*, *IQCB1* and *SPATA7* in one family each, respectively. Six of the ten (60%) mutations identified are novel. Homozygosity mapping using Affymetrix 10K HMA GeneChip on the arRP family identified a novel nonsense mutation in *MERTK*. The mutations segregated within the family and was absent in 200 control chromosomes screened. In one of the eleven LCA families, the causative gene/mutation was not identified but many homozygous blocks were noted indicating that a possible novel locus/gene might be involved. The genotype and phenotype features, especially the fundus changes for *AIPL1*, *RPE65*, *CRB1*, *RDH12* genes were as reported earlier.

## Introduction

Retinal degenerations are the major cause of incurable blindness characterized by loss of photoreceptor cells and present with both genetic and phenotypic heterogeneity [1]. Leber congenital amaurosis (LCA; [OMIM] #204000) is an inherited retinal disease characterized by severe visual loss at birth, nystagmus, and minimal or absent recordable responses on the electroretinogram (ERG) before or by one year of age and accounting for 5% of all retinal dystrophies [2, 3]. LCA presents with variety of fundus changes e.g. the marbleized fundus appearance in *CEP290* gene mutation, RPE atrophy, arteriolar narrowing, pigmentation in fundus in *RPE65* gene involvement, granular pigmentation in *GUCY2D* and peripheral coats like vasculopathy in *CRBI* mutation etc [4]. Some of other clinical findings include high hypermetrophia, oculodigital signs, keratoconus and cataract [3]. The reported prevalence of the disease is 1:50,000–100,000 [5]. Retinitis pigmentosa (RP; [OMIM] #268000) also a retinal degenerative disease, is characterized by progressive degeneration of rods followed by cones, thereby affecting the night and peripheral vision initially and later central vision as well and the classic fundus appearance reveals dark pigmentary clumps in the mid-periphery (“Bone spicules”), attenuated retinal vessels, waxy optic disc pallor. Some of the secondary features of RP include cystoids macular edema, cataract, fine pigmented vitreous cells [6]. The age of onset of RP varies from very early childhood, to sixth decade of life [7] and affects 1 in 2500–7000 individuals in general population [8]. A recent epidemiological survey in Danish population has revealed the prevalence of generalized retinal dystrophies as 1:3454 [9].

LCA is usually inherited as an autosomal recessive disease, but few cases of dominant inheritance are also reported [10]. So far twenty-three genes are implicated in LCA. These candidate genes have been identified by using various methodoliges like, either by candidate gene screening, or linkage studies on large families or homozygosity mapping on nuclear families using either microsatellite markers or SNP microarrays, or screening genes which are involved in retinal function/tissue specific expression, or recently by whole exome seqeuncing [11–15]. These genes account for 70% of LCA cases but the mutation frequency vary among different ethnic populations [16]. The non-syndromic forms of RP are predominantly inherited as either autosomal dominant, autosomal recessive or X-linked recessive, but rarer forms such as X-linked dominant, mitochondrial, and digenic (due to mutations in two different genes) have also been reported [17]. Till date, seventy four loci and sixty seven causative genes have been identified for non-syndromic RP [18] and the prevalence of these known genes varies in different populations [16, 19]. The phenotypic and genetic heterogeneity of LCA, RP and various other inherited retinal dystrophies contribute to complexity of the clinical diagnosis and make molecular testing technically challenging. Also there is considerable overlap between the genotype and phenotype; the same gene may present with different phenotypes in the retinal degenerative disease (RDD) spectrum or the same gene may cause an isolated RDD or systemic disease along with RDD [20].

Identifying the causative gene would be very helpful in confirming the diagnosis at the molecular level, aiding in accurate genetic and reproductive counseling, carrier testing, prenatal testing and also predicting the prognosis of the disease [21]. Recent success in gene replacement therapy for *RPE65* on patients with LCA resulting in slight improvement of vision has opened the possibility and holds promise as potential treatment modality for retinal dystrophies in the future [22–25]. These trials are proof-of-concept for gene transfer as a viable therapy for an entire family of eye diseases, thus proving the essentiality for molecular diagnosis and would be very vital for offering gene based therapies in future.

Homozygosity mapping using SNP arrays or microsatellite markers serves as a powerful tool, where the stretches of homozygous blocks indicate potential candidate gene/locus in autosomal recessive disease [26]. Consanguineous families, populations/communities practicing consanguinity for many generations and geographically isolated populations with inbreed marriages are good candidates for homozygosity mapping because there is an increased percentage of homozygosity in their genome due to identity by descent [27]. Homozygosity mapping has been extensively used to map disease loci for autosomal recessive diseases and there are reports of homozygosity mapping on autosomal recessive RP families from India. [28–30]. We have also previously reported identification of a novel missense mutation leading to activation of cryptic splice site in *LCA5* in a consanguineous LCA family using homozygosity mapping [31]. There are reports from India on mutational screening of few genes for LCA by direct sequencing of the coding regions [32, 33], allele specific ligation assay [34], and APEX chip screening where reported mutations were screened [35]. Homozygosity mapping however adds on to the advantage that along with identifying the reported or novel mutations in the known candidate gene(s), novel loci/genes can also be mapped [36].

Here we have performed homozygosity mapping using Affymetrix 250K and 10K HMA GeneChip in eleven LCA and one arRP consanguineous south Indian families, respectively to know the prevalence of mutations in known genes and to know involvement of novel loci, if any.

## Materials and Methods

### Subjects

Eleven LCA and one arRP consanguineous families were enrolled in the study. Complete ophthalmic examination was carried out for all the affected individuals that included electroretinogram (ERG), fundus photograph, fundus auto fluorescence (FAF) in all patients and optical coherence tomography (OCT) where ever possible. Blood (10ml) was collected from all the affected individuals, unaffected siblings and parents after obtaining written informed consent. The study was approved by the Vision Research Foundation’s Institutional Review Board (IRB) and ethics committee and all the procedures were performed in accordance with institutional guidelines and the Declaration of Helsinki.

### Homozygosity mapping

Genomic DNA was extracted using Nucleospin Blood XL kit (Macherey-Nagel, GmbH, Düren, Germany) according to the manufacturer’s instructions. The eleven LCA families (LCA1-LCA11) were genotyped for 262,000 SNPs using GeneChip Human Mapping 250K NspI Array (Affymetrix, Santa Clara, CA) and the individuals from the arRP family (arRP1) were genotyped for 11,555 SNPs using GeneChip Human Mapping 10K XbaI Array (Affymetrix, Santa Clara, CA) according to the manufacturer’s protocol.

SNP Genotyping was done on one or more affected family members along with an unaffected sibling. Following genotyping using 250K NspI GeneChip, the homozygous regions were analysed using Genotyping Console v4.0 (Affymetrix, Santa Clara, CA). The internal quality control check was set as 90%. Loss of heterozygosity (LOH) score is a measure for the likelihood of a stretch of SNPs to be homozygous based on the population SNP allele frequencies and a score of ≥15 is considered to be significant [37, 38]. Homozygous stretches between the affected and the unaffected were compared by LOH status. The homozygous blocks in the known LCA candidate genes loci and all other homozygous blocks were noted. We first screened the known LCA gene present in the largest homozygous block, followed by others, if required. When the causative mutation was identified, segregation analysis in the family members and control screening was performed to confirm its pathogenicity.

For the arRP1 family, that was genotyped using 10K HMA GeneChip, three affected and one unaffected members were taken for analysis. The internal quality control check was set as 90%. CEL files generated for each sample were analyzed using GTYPE software. The genotype generated was exported to excel sheet for further analysis. Here, the data was first sorted according to chromosome number and then by cytoband position (p arm and q arm). The sorted data was compared between the affected and unaffected for large continuous stretch of homozygous regions (consecutive SNP being homozygous). Chromosomal segments were considered to be homozygous if they had ≥39 consecutive SNPs homozygous since the likelihood of this to occur by chance is 1:100 in consanguineous families [39].

### Mutation Analysis

Primers were designed using Primer 3 (v. 0.4.0) software [40] for the exons along with 50bp of flanking intronic regions for the LCA and arRP candidate genes. The exons were PCR amplified and sequenced using ABI 3100 *Avant* Genetic Analyser (Applied Biosystems, Foster City, CA). Segregation analysis within the family and control screening in 200 chromosomes was performed for the identified mutation(s) by direct sequencing.

### Bioinformatics analysis

The intronic mutations were analysed by Human Splice Finder 2.4.1 [41, 42] and Mutation taster [43] for possible splicing defects and the missense mutations were anlaysed with PolyPhen-2 [44] and SIFT [45] to predict their possible effect on structure and function of the protein.

### RNA extraction and cDNA analysis

Heparin blood samples were allowed to stand at room temperature for one hour and then the buffy coat collected separately. RNA was extracted using Nucleospin RNA II kit (Macherey-Nagel, GmbH, Düren, Germany). The RNA was converted to cDNA using Verso cDNA kit (Fischer Scientific, Surrey, U.K) and the cDNA amplified using specific primers encompassing exons 11, 12, and 13 of *IQCB1* gene.

## Results

### Molecular Diagnosis

We performed the homozygosity mapping for the eleven LCA families with 250K HMA GeneChip on 32 individuals of which 23 were affected and 9 unaffected siblings and with 10K HMA GeneChip for three affected individuals and one unaffected individual in the arRP family. In each of the LCA family, we were able to identify on an average of about fifteen homozygous blocks ranging in size from 1Mb to 33 Mb. Out of eleven LCA families, we identified the causative mutation in ten families (90%), *AIPL1* mutation in three, *RPE65* mutation in two, and *CRB1*, *GUCY2D*, *IQCB1*, *RDH12*, *SPATA7* mutation in one family each, respectively. In the arRP family, we identified a novel nonsense mutation in *MERTK*. Table 1 shows the list of known LCA candidate genes present within the homozygous blocks in each LCA and arRP family. Table 2 shows the total number of homozygous blocks >1Mb in the LCA and arRP families. Table 3 lists the mutations identified in LCA and arRP families. All the novel mutations are submitted in Leiden Open Variation Database (LOVD) v.3.0 [46].

**Table 1:** Size of homozygous blocks and the known LCA candidate genes identified in LCA families and the arRP family.

S.No	Family ID	Number of Affected individuals taken for analysis	Size of the homozygous block in which known candidate gene(s) were present (Mb)	Chromosome location	Genes Screened	Gene reference ID
1.	LCA-1	2	13	1p31.3	*RPE65*	NM_000329.2
2.	LCA-2	4	26	1q31.3	*CRB1*	NM_001257965.1
3.	LCA-3	2	3	17p31.1	*GUCY2D*	NM_000180.3
4.	LCA-4	2	4.7	3q13.3	*IQCB1*	NM_001023570.2
5.	LCA-5	2	3.7	17p13.2	*AIPL1*	NM_014226.3
6.	LCA-6	2	4.05	14q11.2	*RPGRIP1*	NM_020366.3
	LCA-6	2	1	2q13	*MERTK*	NM_006343.2
7.	LCA-7	2	6	14q11.2	*RPGRIP1*	NM_020366.3
	LCA-7	2	1.3	14q24.1	*RDH12*	NM_152443.2
8.	LCA-8	1	5	17p13.2	*AIPL1*	NM_014226.3
9.	LCA-9	2	30	1p31.3	*RPE65*	NM_000329.2
10.	LCA-10	2	4.9	17p13.2	*AIPL1*	NM_014226.3
11.	LCA-11	2	6	14q31.3	*SPATA7*	NM_018418.4
12.	arRP 1	3	1.1	2q13	*MERTK*	NM_006343.2

**Table 2:** Total number of homozygous blocks ≥ 1Mb in the LCA and arRP families.

S.No	Family ID	Number of ≥1Mb blocks
1.	LCA-1	8
2.	LCA-2	7
3.	LCA-3	15
4.	LCA-4	8
5.	LCA-5	20
6.	LCA-6	16
7.	LCA-7	33
8.	LCA-8	28
9.	LCA-9	9
10.	LCA-10	15
11.	LCA-11	18
12.	arRP 1	1

**Table 3:** Mutations identified in LCA families and the arRP family.

S.No	Family ID	Genes Screened	Exon/intron	Mutation identified	Predicted change in protein	Effect of identified sequence variations
1.	LCA-1	*RPE65*	intron 8	c.858+1G>T Reported [55]	(r.spl?)	Pathogenic
2.	LCA-2	*CRB1*	Exon 9	c.3307G>A Reported [47, 56]	p.(Gly1103Arg)	Pathogenic
3.	LCA-3	*GUCY2D*	Exon 3	c.994delC **Novel**	p.(Arg332Alafs*63)	Pathogenic
4.	LCA-4	*IQCB1*	intron 12	c.1278+6T>A **Novel**	r.[1131_1278 del,1131_1278del] p.(Gln378Alafs*2)	Pathogenic
5.	LCA-5	*AIPL1*	Exon 6	c.834G>A Reported [35, 53, 57–60]	p.(Trp278*)	Pathogenic
6.	LCA-6	*RPGRIP1 MERTK*	-	Not identified	-	-
7.	LCA-8	*AIPL1*	Exon 2	c.247G>A **Novel**	p.(Glu83Lys)	Pathogenic
8.	LCA-9	*RPE65*	Exon 13	c.1409C>TReported [32]	p.(Pro470Leu)	Pathogenic
9.	LCA-10	*AIPL1*	Exon 4	c.613_622 delATCATCTGCC **Novel**	p.(Ile205*)	Pathogenic
10.	LCA-11	*SPATA7*	intron 7	c.913-2A>G **Novel**	(r.spl?)	Pathogenic
11.	arRP1	*MERTK*	Exon 4	c.721C>T **Novel**	p.(Gln 241*)	Pathogenic
12.	LCA-7	*RPGRIP1R DH12*	RDH12 intron 3	c.344-8C>T**Novel**	(r.spl?)	Possibly pathogenic

**Fig 1:**
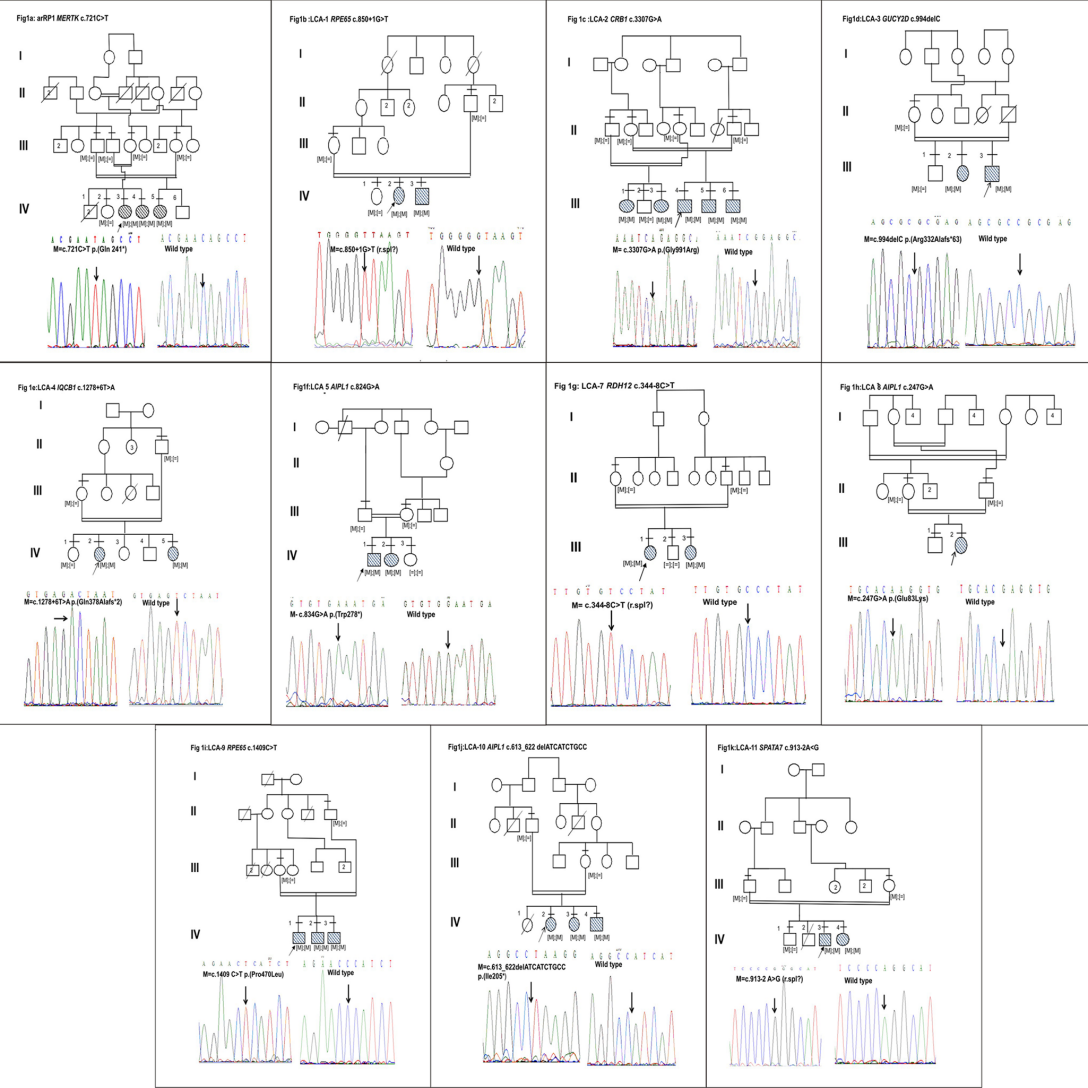
Segregation analysis. 1a:arRP1 *MERTK* c.721C>T, 1b:LCA-1 *RPE65* c.850+1G>T, 1c: LCA-2 *CRB1* c.3307G>A, 1d:LCA-3 *GUCY2D* c.994delC, 1e:LCA-4 *IQCB1* c.1278+6T>A, 1f:LCA-5 *AIPL1* c.824G>A, 1g: LCA-7 *RDH12* c.344-8C>T, 1h:LCA-8 *AIPL1* c.247G>A, 1i:LCA-9 *RPE65* c.1409C>T, 1j:LCA-10 *AIPL1* c.613_622 delATCATCTGCC, 1k:LCA-11 *SPATA7* c.913-2A>G. The arrow indicates the index case. The filled in circles and squares are affected females and males respectively. [M];[M]–affected with homozygous mutation, [M]; [=] –carries for any given mutation and [=]; [=] –wild type. Lines above the individual indicate availability of genotype.

Segregation analysis (Fig 1a–1k) was performed in all the families and the mutation segregated within the family, with all the affected being homozygous for mutation, parent(s) being heterozygous carriers and the unaffected being either heterozygous for mutation or wild type. One hundred normal controls (200 chromosomes) were screened for the identified mutations and all were wild type.

**Fig 2:**
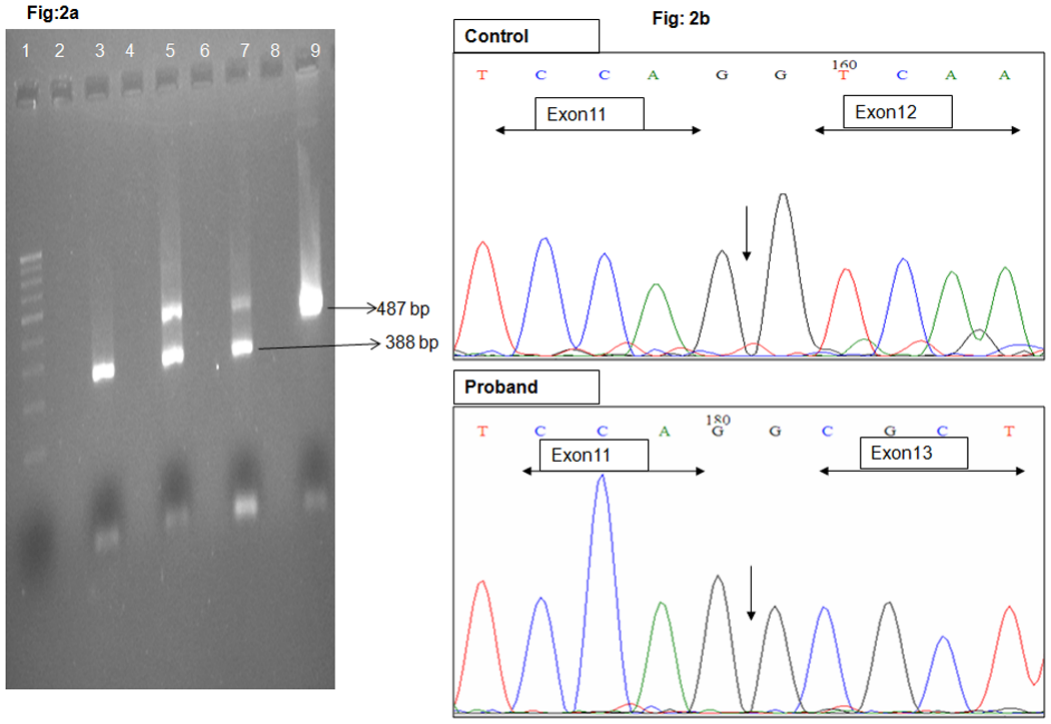
2% Agarose gel electrophoresis showing cDNA amplification of exon 11–13 of *IQCB1*. Lane 1-100bp ladder, Lane 3- Affected index case, Lane 5 & 7—Carrier parents, Lane 9—Control, Lane 2, 4, 6, 8—empty wells Fig 2b Eletrophoretogram trace showing the amplified cDNA of control and proband. In proband exon 11 is followed by exon 13 and exon 12 is completely deleted, whereas in control, exon 11, 12 and 13 is continuous. The end of exon 11 is marked in both the phoretograms.

The four intronic mutations; *RPE65* c.858+1G>T, *SPATA7* c.913-2A>G, *IQCB1* c.1278+6T>A, *RDH12* c.344-8C>T, are present either in the conserved splice acceptor or donor site or within ten bases of the intron following the exon. Analysis of cDNA prepared from lymphocytes of LCA-4 family members using specific primers encompassing *IQCB1* exons 11–13, revealed a single transcript of 338bp in the affected and two transcripts of 338 and 487bp, respectively in the heretozygous carrier parents (Fig 2a). Direct sequencing revealed that in the proband, exon 12 has been completely deleted (338bp amplicon) whereas both the parents were heterozygous carriers (338bp deleted and 478 wild type amplicons, respectively; Fig 2b). This skipping of exon 12 in the affected is predicted to result in a truncated protein, p.(Gln378Alafs*2). The consequence of c.344-8C>T mutation on the splicing of the retinal-specific *RDH12* gene could not been determined but the change was predicted to alter splicing. The two missense mutations, *CRB1* c.3307G>A p.(Gly1103Arg) and *AIPL1* c.247G>A p.(Glu83Lys) analysed with PolyPhen 2 [44] and SIFT [45] were both predicted to be probably damaging and deleterious (Table 4 details the results of the in-silico analysis).

**Table 4:** Probable effects of splice site mutations using HSF 2.4.1 and Mutation Taster and effects of missense mutations using PolyPhen and SIFT.

S.No	Family ID	Gene	Mutation identified	HSF Wild type Consensous value (CV)	HSF Mutant consensous value (CV)	HSF delta CV (%)	MT Wild type scoring	MT Mutant scoring	MT Splice site change	cDNA Analysis	PolyPhen score	Polyphen predicted effect	SIFT score	SIFT Predicted effect
1.	LCA-1	*RPE65*	c.858+1G>T	-	-	-	-	-	Likely to Disturb Normal Splicing, sequence motif lost	**Not done**	NA	NA	NA	NA
2.	LCA-4	*IQCB1*	c.1278+6T>A	79.28	75.78	-4.42	0.36	0.95	Donor increased	r.[1131_1278 del,1131_1278del]**Exon12 skipping**	NA	NA	NA	NA
3.	LCA-7	*RDH12*	c.344-8C>T	73.62	70.25	-4.57	0.55	0.76	Acceptor increased	**Not done**	NA	NA	NA	NA
4.	LCA-11	*SPATA7*	c.913-2A>G	86.72	57.09	Site broken	0.53	0.84	Acceptor increased	**Not done**	NA	NA	NA	NA
5.	LCA-2	*CRB1*	c.3307G>Ap.(Gly1103Arg)	NA	NA	NA	NA	NA	NA	NA	0.91	Probably damaging	0	Deleterious
6.	LCA-8	*AIPL1*	c.247G>A p.(Glu83Lys)	NA	NA	NA	NA	NA	NA	NA	1.0	Probably damaging	0	Deleterious

NA-not applicable

The human splice finder presents consensous value (CV) which indicate strength of the splice site range from 0 to 100. The splice sites of CV higher than 80 are considered as strong splice sites, 70–80 as less strong and 65–70 as weak, and a CV below 70 is considered to be non-functional [41]. The mutation taster (MT) scores the wild type and the mutant and a confidence score of >0.3 for the mutant indicates gain of completely new splice site [43].

### Genotype-Phenotype correlation for the mutations identified in LCA

In this study, mutations in *AIPL1* and *RPE65* were identified in three and two families, respectively. Whereas mutations in other genes namely, *GUCY2D*, *CRB1*, *IQCB1*, *RDH12*, *SPATA7* were identified in one family each.

#### Patients with AIPL1 mutations

**Fig 3:**
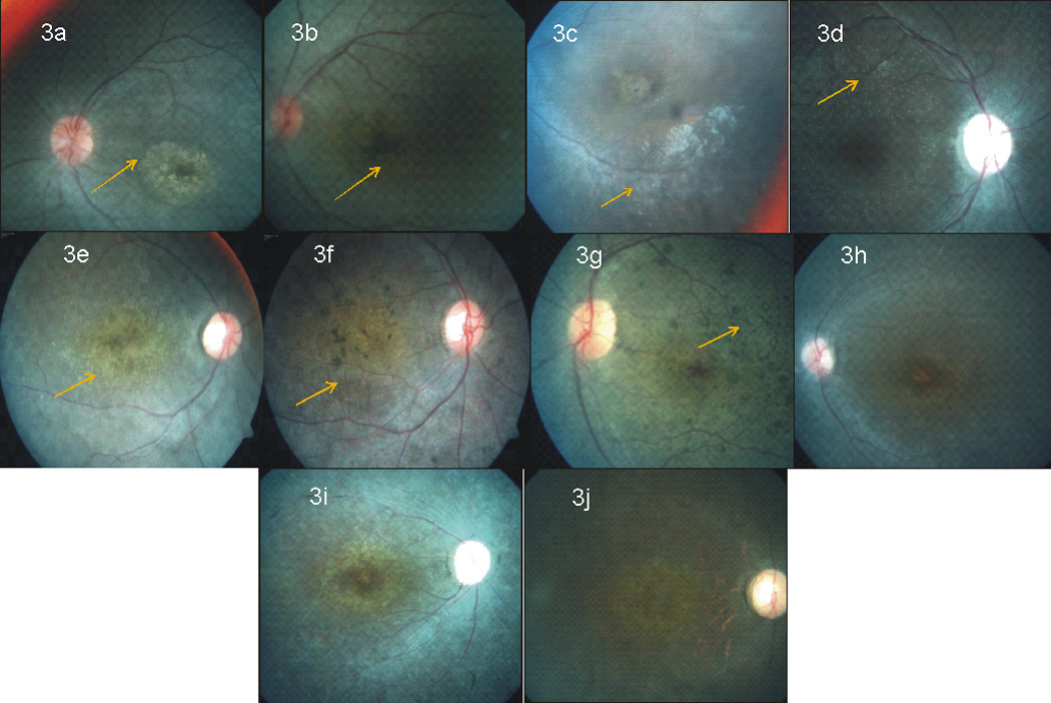
Fundus photographs. Fig 3a A 10yrs old female with c.824G>A p.(Trp278*) mutation in *AIPL1* (LCA-5 family) showed normal disc, attenuated vessels, (arrow mark indicates) yellow patches in macula. Fig 3b A 14yrs old male with c.824G>A p.(Trp278*) mutation in *AIPL1* (LCA-5 family, elder sibling) showed normal disc, attenuated vessels, (arrow mark indicates) black pigments in macula. Fig 3c A 18 yrs old female with c.850+1G>T (r.spl?) mutation in *RPE65* (LCA-1 family) showed pallor disc, attenuated vessels with scar in the macula, peripheral RPE mottling (marked with arrow) Fig 3d A 28yrs old male with c.1409C>T p.(Pro470Leu) mutation in *RPE65* (LCA-9 family) showed pallor disc, attenuated vessels, normal macula, with salt and pepper fundus. Arrow mark shows distinct pin head size yellow white dot like spots at the posterior pole. Fig 3e A 14 yrs old female with c.2971G>A p.(Gly991Arg) mutation in *CRB1* (LCA-2 family) showed coin shaped pigment clumps and greyish atrophic changes seen in the macula, (arrow mark indicates the macula) Fig 3f A 18 yrs old female with c.2971G>A p.(Gly991Arg) mutation in *CRB1* (LCA-2 family, elder sibling) showed pale disc, attenuated vessels, atrophic macula with nummular pigment clumps and greyish atrophic reflex (arrow mark indicates the macula) Fig 3g A 19yrs old male female with c.2971G>A p.(Gly991Arg) mutation in *CRB1* (LCA-2 family, eldest sibling) showed coin shaped pigment clumps seen in the background (arrow mark indicates the coin shaped clumps) All the three affected siblings show progressive changes in macula with age for *CRB1* mutation positive family. Fig 3h, 3i, 3j A 24 yrs old female, a 25 yrs old female and a 32 yrs old female with c.721C>T p.(Gln 241*) mutation in *MERTK* (arRP1 family) showing mild, milder and marked features of RP, respectively. Progressive changes with age in the macula are observed.

In the three families, three different types of mutations were observed, a reported nonsense mutation, c.834G>A p.(Trp278*) in family LCA-5, a novel missense mutation, c.247G>A p.(Glu83Lys) in family LCA-8 and a novel 10-base pair deletion, c.613_622delATCATCTGCC p.(Ile205*) in family LCA-10. In LCA-5 family, both the affected siblings had normal disc, and mildly attenuated vessels. Yellowish atrophic patches were seen in the macular area in the younger sibling (10 yrs) (Fig 3a), while the elder sibling (14yrs) had atrophic macula with black pigments (Fig 3b). Both the siblings had salt and pepper fundus with bony spicules. In LCA-8 family there was only one affected person phenotyped at the age of 5 years, with normal disc, mildly attenuated vessels, and atrophic macula with peripheral RPE granularity. In LCA-10 family, the three affected siblings also had atrophic macular degeneration with bony spicules, attenuated vessels, all seen in their third decade of life. The cases in the genotyped families revealed three different mutations but were similar phenotypically with severity of the retinal changes increasing with age reflecting the progressive nature of the disease and macular degeneration being a characteristic feature in *AILP1* mutation positive LCA cases.

#### RPE65

In two families, we identified *RPE65* mutation, a reported splice site mutation, c.858+1G>T (r.spl?) in family LCA-1 and a reported missense mutation, c.1409C>T p.(Pro470Leu) in family LCA-9. In LCA-1 family, both the affected siblings phenotyped in their second decade had pale disc with attenuated vessels, salt and pepper fundus with peripheral RPE mottling. The elder sibling also revealed macular scarring (Fig 3c) and the younger sibling had very few early alterations in the macula. In LCA-9 family, all the three affected siblings phenotyped in their third decade had pale disc, attenuated vessels, normal macula with salt and pepper appearance in the periphery. In both the families affected individuals had profound visual loss. The eldest sibling (28yrs) of LCA-9 family also showed presence of distinct pin head sized white spots at the posterior pole (Fig 3d).

#### CRB1

In family LCA-2 with four affected members, a reported missense mutation, c.2971G>A p.(Gly991Arg) was identified in *CRB1*. All the four affected members in their second decade had profound visual loss and all had a typical fundus picture of pale disc, para-arteriolar preservation of the retinal pigment epithelium (PPRPE), and atrophic macula with nummular pigment clumps and greyish atrophic reflex along with coin shaped pigment clumps seen at the background (Fig 3e, 3f and 3g).

#### GUCY2D

In family LCA3 with two affected members, a novel frameshift mutation, c.994delC p.(Arg332Alafs*63) was observed in *GUCY2D*. Both the siblings in their late teenage had profound visual loss and showed fundus picture of pale disc, minimal arteriolar attenuation and normal looking macula.

#### IQCB1

A novel *IQCB1* splice mutation, c.1278+6T>A r.[1131_1278 del,1131_1278del] p. (Gln378Alafs*2) was seen in LCA-4 family. The two affected siblings showed pale disc, attenuated vessels, normal macula and plenty of hypo-pigmented lesion, tapetal reflex was seen at the background in the elder sibling (34y), whereas the younger sibling (27yrs) had little hypo-pigmentation.

#### RDH12

Family LCA-7 had a novel possible pathogenic variant in *RDH12*, c.344-8C>T (r.spl?). The two affected siblings, one aged 26yrs and other 10yrs showed normal disc, attenuated arteriolar vessels, and macula revealed small horizontal oval area (bull’s eye like lesion) along with metallic sheen in the background. Atrophic changes in the macula were seen in the first decade itself in the younger sibling.

#### SPATA7

A novel spice site mutation, c.913-2A>G (r.spl?) was seen in family LCA-11, with two affected siblings aged 8yrs and 2yrs. Fundus picture of both the siblings revealed presence of mild disc pallor, arteriolar attenuation and peripheral RPE mottling.

#### Phenotype of the MERTK mutation positive arRP family

In arRP1 family there were three affected siblings, two were in their second decade of life and the eldest sister was in her third decade of life. Fundus picture revealed pallor disc, marked attenuated vessels, atrophic macula, bone spicule pigment and widespread RPE atrophy. This family is marked by a progressive change in the fundus (Fig 3h, 3i and 3j).

The clinical details; refraction, visual acuity, nystagmus, ERG and fundus details of all the affected individuals are given in S1 Table.

## Discussion

In our study, of eleven consanguineous LCA families analysed by homozygosity mapping followed by candidate gene screening, we identified the causative mutations in ten families (90%). Also we identified the causative gene and mutation in one arRP family studied. Homozygosity mapping involves detecting the disease loci by exploiting the fact that the adjacent region i.e short chromosomal segments surrounding the homozygous mutation had not been crossed over and the surrounding single nucleotide polymorphism (SNP) would also be in a homozygous state and these regions would be inherited by descent (Identical by descent) from a common ancestor [47]. For most of our families the largest homozygous blocks carrying the disease gene fell within the first three positions but there were also some exceptions, where the disease allele was not among largest homozygous blocks.

The significant homozygous blocks were in the average size from 1Mb to about 33Mb, differing for each family and harboring the candidate LCA gene(s). Also, when more number of affected members were genotyped, the number of homozygous blocks shared among the affected was less, enabling easier identification of the candidate locus/gene.

There were six novel mutations in six LCA families and one novel mutation in the arRP family identified in this study; *AIPL1-*2 mutations, and one each in *GUCY2D*, *IQCB1*, *RDH12*, *SPATA7*, and *MERTK* (in arRP family). Of the ten mutations identified in the eleven LCA families, two are splice site mutations, one each in *RPE65*, and *SPATA7* and two intronic mutations within 10bp of the intron in *IQCB1* and *RDH12*, respectively. These splice site mutations analysed with bioinformatics tools, HSF2.4.1 and Mutation taster 2 were predicted to result in loss of splicing, whereas the mutations within 10bp of the intron in *RDH12* and *IQCB1* were predicted to activate cryptic splice site. We performed cDNA analysis for the *IQCB1* mutation, as this gene is expressed in lymphocyte as well. *IQCB1* gene encodes for nephrocystin protein which interacts with calmodulin and retinitis GTPase regulator protein. Defects in this gene are reported for Senior-Loken Syndrome type 5 [48]. Splice site mutation has been previously reported in a nephronophthisis patient and also in two LCA families [49, 50]. In a study done by Estrada-Cuzcano et al [51], eleven *IQCB1* mutations were identified in a cohort of 150 LCA patients. During revaluation, seven of the mutation positive cases were found to have developed renal complications, thus re-diagnosed to have Senior-Loken Syndrome, while rest of the four patients reported no kidney abnormalities but they had similar mutations found in nephronophthsis patients. In our cohort, the family LCA-4, with two affected siblings (sisters) was initially diagnosed with LCA and reported no renal abnormalities. However, when we recalled the family for cDNA analysis after identification of the mutation, the family reported that the proband now 34 years had a sudden onset of renal failure (both the kidneys) at the age of 31 years and is under treatment. We could perform the cDNA analysis in the younger affected sibling and the carrier parents only, and till now the younger sibling (29y) has no renal complications. cDNA analysis confirmed that the mutation, c.1278+6T>A activates cryptic splice site leading to complete skipping of exon 12 resulting in a predicted truncated protein p.(Gln378Alafs*2). *IQCB1* mutation positive LCA patients may be at risk of developing renal abnormalities, however the onset of the renal failure is highly variable [51] and need to be counseled and managed appropriately. cDNA analysis for *RDH12*, c.344-8C>T mutation could not be done because the gene is not expressed in lymphocytes and has exclusive retinal expression. However, we consider this to be a possible pathogenic variant which might be causative for the disease phenotype; a) through homozygosity mapping we identified two large homozygous blocks spanning about 6Mb and 1.3 Mb containing two known LCA candidate genes, the larger block had *RPGRIP1* and the smaller block had *RDH12*. Firstly, screening *RPGRIP1* did not reveal any pathogenic variant, hence it was followed by screening *RDH12*, where we found the intronic variant, c.344-8 C>T, which segregated with disease phenotype in the family and was absent in 200 control chromosomes screened. b) *in-silco* analysis predicted the variant to activate the cryptic splice site affecting/altering the protein and c) phenotypically the fundus of both the affected sibs too showed maculopathy in the first decade of life, a feature previously observed in *RDH12* mutation positive cases. [52].

### Genotype-Phenotype correlation

In patients with *AIPL1* mutations (three) atrophic macula and bony spicules were common features, as reported earlier [4, 53]. While fine pigments were seen in the periphery only in elder patients but these were not seen in younger patients. Patients with *RPE65* mutation showed tapetal reflex, disc pallor, attenuated vessels, typical bony spicules with salt and pepper fundus and normal macula as described earlier [16]. Distinct yellow white dot like lesion appeared in eldest member of LCA-9 family as well as in the other family who had the same mutation from our previous study [32] (Fig 3d). Whether these particular RPE white dots are specific to this particular type of missense mutation or for mutations only in exon 13 is not known. In *CRB1* mutation positive siblings showed typically described mild para-arteriolar preservation of the retinal pigment epithelium (PPRPE) in their fundus [54] along with coin shaped pigment clumps at the background. In *RDH12* mutation positive patients too, pronounced maculopathy and bony spicules were observed as reported [52]. *GUCY2D* mutation positive patients showed normal macula and vessels and *SPATA7* mutation positive patients showed mild disc pallor, arteriolar attenuation and peripheral RPE mottling.

### Mutation negative family

In one family (LCA-6) we were unable to identify the causative gene/mutation in the known LCA candidate gene(s); there were two homozygous blocks with known LCA genes, *RPGRIP1* and *MERTK*. These two did not harbor any pathogenic mutation, however there were fourteen other homozygous blocks shared between the affected and ranging in size from 1-7Mb with no known LCA candidate genes. The clinical details of the affected members of this family are given in S1 Table. Homozygosity mapping has revealed many homozygous blocks and the causative gene/mutation which may either be a novel gene or a gene involved in other retinal disease is most likely to be present in one of these blocks. We have however not screened the intronic and regulatory regions of the known candidate gene(s) in the family and hence cannot rule out the possibility of deep intronic mutations or mutations in regulatory regions that might be pathogenic. Nevertheless, homozygosity mapping has helped in indicating possible novel disease locus.

## Conclusion

In our study, we performed the homozygosity mapping using 250K and 10K Affymetrix GeneChip for eleven consanguineous LCA families and one consanguineous arRP family, respectively. We were able to identify the mutations or likely disease-causing mutations in ten LCA and one arRP families (11 out of 12) (90%). Of the mutations identified 58% (7 out of 12) were novel involving seven different genes for LCA. The molecular diagnosis has not only confirmed the genetic heterogeneity and certain specific phenotypic features aiding in prognosis prediction but also helped in appropriate counseling. Absence of mutation in known candidate gene in one LCA family indicate involvement of further novel gene(s) in the disease.

## Supporting Information

S1 Table The clinical details; refraction, visual acuity, nystagmus, ERG and fundus details of all the affected individuals. (XLS)
